# Hysteroscopy as a standard procedure for assessing endometrial lesions among postmenopausal women

**DOI:** 10.1590/S1516-31802007000600007

**Published:** 2007-11-01

**Authors:** Camila Toffoli Ribeiro, Júlio César Rosa-e-Silva, Marcos Felipe Silva-de-Sá, Ana Carolina Japur de Sá Rosa-e-Silva, Omero Benedicto Poli Neto, Francisco José Candido dos Reis, Antonio Alberto Nogueira

**Affiliations:** 1 Department of Gynecology and Obstetrics, Faculdade de Medicina de Ribeirão Preto (FMRP), Universidade de São Paulo (USP), São Paulo, Brazil

**Keywords:** Hysteroscopy, Ultrasonics, Postmenopause, Biopsy, Endometrial carcinoma, Histeroscopia, Ultra-som, Pós-menopausa, Biópsia, Neoplasias do endométrio

## Abstract

**CONTEXT AND OBJECTIVES:**

Endometrial cancer is the most prevalent type of malignant neoplasia of the genital tract. The objective of this study was to calculate the sensitivity, specificity, accuracy and positive and negative predictive values for diagnostic hysteroscopy, in comparison with histopathological tests, for all lesions of the endometrial cavity.

**DESIGN AND SETTING:**

Retrospective descriptive study at the public tertiary-level university hospital of Faculdade de Medicina de Ribeirão Preto, Universidade de São Paulo.

**METHODS:**

Diagnostic hysteroscopy was indicated in the following instances: endometrial thickness > 4 mm in asymptomatic patients; postmenopausal bleeding; and irregular endometrium or endometrium difficult to assess from ultrasound, with or without vaginal bleeding. Ultrasound evaluations were carried out no more than three months prior to hysteroscopy.

**RESULTS:**

There were 510 patients, with a mean age of 61.1 ± 2.0 years and mean time elapsed since the menopause of 12.7 ± 2.5 years. Endometrial biopsies were performed on 293 patients (57.5%). Histopathological analysis showed that 18 patients presented endometrial carcinoma or typical or atypical hyperplasia, and none of them presented endometrial thickness of less than 8 mm. No significant differences were found between the median thicknesses of the various benign lesions (p > 0.05). In our data, the sensitivity, specificity, accuracy and positive and negative predictive values for cancer or hyperplasia were 94.4%, 97.0%, 96.8%, 68% and 99.6%, respectively.

**CONCLUSIONS:**

Our results suggest that hysteroscopy is valuable as a diagnostic tool for malignant/hyperplastic and benign lesions, except for submucous myomas, for which the sensitivity was only 52.6%.

## RESUMEN

**CONTEXTO E OBJETIVO:**

O câncer endometrial é o tipo mais prevalente de neoplasia maligna do trato genital. Os objetivos deste estudo foram: calcular a sensibilidade, especificidade e acurácia, bem como valor preditivo positivo e negativo das histeroscopias diagnósticas em comparação com a análise histopatológica de todas as lesões da cavidade endometrial.

**DESENHO E LOCAL:**

Estudo retrospectivo e descritivo no setor de endoscopia ginecológica do hospital universitário, terciário e público da Faculdade de Medicina de Ribeirão Preto, Universidade de São Paulo.

**MÉTODOS:**

A histeroscopia diagnóstica foi indicada nas seguintes situações: espessura endometrial > 4 mm em pacientes assintomáticas, sangramento na pós-menopausa, endométrio irregular ou endométrio de difícil avaliação pelo ultra-som, com ou sem sangramento vaginal. A avaliação ultra-sonográfica foi realizada não mais que três meses antes da histeroscopia.

**RESULTADOS:**

A idade média das pacientes foi 61,1 ± 2,0 anos, e a duração média do período pós-menopausa foi de 12,7 ± 2,5 anos. Das 510 pacientes, 293 (57,5%) foram submetidas à biópsia endometrial, no estudo histopatológico, 18 pacientes apresentavam carcinoma endometrial, hiperplasia típica ou atípica e nenhuma delas apresentava espessura endometrial maior que 8 mm. Nenhuma diferença significativa foi encontrada entre as espessuras medianas das várias lesões benignas (p > 0,05). A sensibilidade (94,4%), especificidade (97%), acurácia (96,8%) e valores preditivos positivo e negativo (68% e 99,6% respectivamente) foram altos em nosso estudo.

**CONCLUSÃO:**

Nossos resultados sugerem que a histeroscopia diagnóstica apresenta boa validade como ferramenta diagnóstica para lesões malignas e hiperplasias, bem como para lesões benignas, com exceção dos leiomiomas submucosos, para o qual a sensibilidade foi de somente 52,6%.

## INTRODUCTION

Endometrial cancer, the most prevalent type of malignant neoplasia of the genital tract in Western countries,^1^ also presents the best prognosis, since approximately 70% of the cases are detected in their early stages.^2^ Between 80 and 95% of such patients present vaginal bleeding as the first symptom,^3^ thus enabling prompt diagnosis and therapeutic intervention.

Because of the early manifestation of symptoms of this neoplasia, the need for a screening program is debatable. One of the most widely investigated tests used for this purpose is transvaginal ultrasound. Despite the fact that some authors have defended its application to asymptomatic patients,^4,5^ others have failed to show any benefits from this procedure, since it appears to present a positive predictive value of only 2%.^6-8^

Nevertheless, since vaginal bleeding is a common symptom among postmenopausal women, ultrasound has been a useful screening method for determining which symptomatic patients should undergo invasive collection of endometrial samples. In this situation, endometrial thickening, as measured by ultrasonography, correlates better with the presence of endometrial lesions^9-11^ than among asymptomatic women.

Depending on the availability of each type of procedure, biopsies can be performed using either image guidance techniques, such as hysteroscopy with direct viewing of the uterine cavity, or various random sample collection methods, such as Pipelle endometrial sampling, Vabra catheter aspiration, Novak curettage or traditional uterine curettage.

Diagnostic hysteroscopy plays a major role in assessing bleeding among postmenopausal women due to its high sensitivity and specificity for diagnosing endometrial lesions.^12-14^ This method has increasingly been used as an alternative to uterine curettage,^15,16^ with the advantage of enabling directed or guided biopsies of small lesions.

## OBJECTIVES

The primary objective of this study was to calculate the sensitivity, specificity, accuracy, positive and negative predictive values and likelihood ratios for diagnostic hysteroscopy, in comparison with histopathological tests, for all lesions of the endometrial cavity. The secondary objective was to determine the mean thicknesses of benign lesions of the endometrial cavity, comparing them to those found in cases of endometrial carcinoma and endometrial hyperplasia.

## MATERIAL AND METHODS

### Type of study

This was a retrospective descriptive study.

### Setting

It was carried out at the public tertiary-level university hospital of Faculdade de Medicina de Ribeirão Preto of Universidade de São Paulo.

### Subjects

The study reviewed the results from 510 diagnostic hysteroscopy procedures that were performed in the gynecological endoscopy clinic of the university hospital between January 2002 and March 2004. The inclusion criteria were that the patients had to have presented amenorrhea for at least one year, and not have taken hormone replacement therapy within the preceding six months.

### Procedures

Diagnostic hysteroscopy was indicated in the following instances: endometrial thickness > 4 mm, as measured by ultrasound in asymptomatic patients; postmenopausal bleeding (the duration of bleeding was defined as the length of time, in months, from the detection of symptoms until the surgery for staging); and irregular endometrium or endometrium that was difficult to assess on ultrasound, with or without vaginal bleeding.

Ultrasound evaluations were carried out no more than three months prior to hysteroscopy, using a 5 to 7.5 MHz transducer and the HDI^®^ 3000 imaging apparatus (high definition imaging; Advanced Technology Laboratories [ATL] Ultrasound, Bothell, Washington, United States). On these ultrasound images, which encompassed a longitudinal section of the uterus, including both endometrial layers and excluding the surrounding hypoechoic halo, the greatest endometrial thickness obtained was recorded. When there was a liquid film between the two endometrial layers, they were measured separately and the values were summed.

All the hysteroscopy procedures were performed in outpatient settings and most were performed without anesthetic, which was only administered if the patient requested it because of pain during the examination. A Storz^®^ optical hysteroscope with a 5 mm diameter (Karl Storz Endoscopy America, Culver City, California, United States) was used. To distend the uterine cavity, carbon dioxide was introduced at a flow rate of 50 ml/min and a maximum pressure of 200 mmHg. When indicated, biopsies were carried out under hysteroscopic viewing. For focal lesions covering less than 25% of the endometrial cavity, Storz^®^ 2 mm biopsy forceps were used, whereas Novak curettes were used for diffuse lesions. The material was fixed in 10% formalin, prepared for paraffin embedding and stained with hematoxylin and eosin (H&E), after which it was sent for histological analysis.

Even when presenting normal hysteroscopic results, patients with endometrial thickening and bleeding underwent Novak curette biopsies.

### Statistical methods

The statistical analysis was conducted using the GraphPad Prism 4.0^®^ 32-bit executable software (GraphPad Software Inc., San Diego, CA, USA). The Mann-Whitney or Kruskal-Wallis test, together with Dunn's post-test, were used for variables that did not follow normal distribution or presented an F test with p < 0.05. The unpaired Student's t test was applied for variables with normal distribution. The significance level was set at p < 0.05.

This study was approved by the Research Ethics Committee of our institution.

## RESULTS

The mean age of the patients was 61.1 ± 2.0 years and the mean time elapsed since the menopause was 12.7 ± 2.5 years. Among the 510 patients studied, 12 were undergoing treatment with tamoxifen. The indications for hysteroscopy are presented in Table 1.

**Table 1:** Indications for diagnostic hysteroscopy among 510 procedures in an university hospital

Indication	n	%
Asymptomatic endometrial thickening	399	78.2
Bleeding with endometrial thickening	58	11.4
Endocervical polyps	17	3.3
Bleeding without thickening	11	2.2
Other	22	4.3
Heterogeneous/poorly-defined endometrium	03	0.6
**Total**	**510**	**100**

Of these 510 patients, 293 (57.5%) underwent endometrial biopsies, of which seven (2.4%) produced a quantity of material that was insufficient for histopathological analysis. None of the remaining 217 patients (42.5%) underwent biopsies, since their uterine cavities were normal and did not present any suspicious areas.

The histopathological analysis showed that 18 patients presented endometrial carcinoma or typical or atypical hyperplasia, or both. None of them presented endometrial thickness of less than 8 mm.

No significant differences were found between the median thicknesses of the various benign lesions (p > 0.05). However, the median thicknesses of endometria and benign lesions differed significantly from the median thicknesses of carcinomas and hyperplasias (p < 0.05 for myomas and functional endometria; p < 0.01 for polyps and endometrial atrophy).

Table 2 correlates the diagnostic hysteroscopy findings with the histopathological findings. The diagnosis based on hysteroscopy results was erroneous in only one case of atypical hyperplasia, which was seen as being consistent with an endometrial polyp (presenting typical vessels).

**Table 2:** Results from diagnostic hysteroscopy compared with the histopathological examination among 510 procedures performed in an university hospital

Hysteroscopy	Anatomopathology n (%)						
	Active or normal endometrium	Polyp	Atrophy	Hormonal imbalance	Cancer/hyperplasia	Myoma	Other
Polyps (n = 193)	13	151	10	2	1	8	8
Myomas (n = 21)	4	4	2	–	–	10	1
Cancer/hyperplasia (n = 25)	1	4	1	2	17	–	–
Normal (n = 13)*	8	–	4	–	–	1	–
Other (n = 34)	4	4	5	2	–	–	19

* Nine cases of hysteroscopy-suggested atrophy and four cases of active endometrium.

The sensitivity, specificity, accuracy, positive and negative predictive values and likelihood ratios for various endometrial cavity abnormalities are listed in Table 3. Cases with biopsy samples that were insufficient or unrepresentative were excluded from the calculations.

**Table 3:** Sensitivity, specificity, accuracy, positive and negative predictive values and likelihood ratios for hysteroscopy in comparison with the histopathological diagnosis

	Sensitivity(%)	Specificity (%)	PPV (%)	NPV (%)	Accuracy (%)	Likelihood ratio positive test	Likelihood ratio negative test
Polyps	92.6	65.8	78.2	86.1	81.1	2.7	0.1
Myomas	52.6	95.9	47.6	96.6	93.0	12.8	0.5
Cancer or hyperplasia	94.4	97.0	68.0	99.6	96.8	32.5	0.05
Normal	35.3	99.6	92.3	91.9	92.0	88.0	0.6

PPV = positive predictive value; NPV = negative predictive value.

Among the symptomatic patients for whom hysteroscopy did not detect any lesions and who then underwent Novak curettage biopsies, there were no cases of biopsy-diagnosed carcinoma or hyperplasia.

Only two asymptomatic patients were diagnosed with carcinoma or endometrial hyperplasia. In the first of these cases, the patient presented complex hyperplasia with atypia, was treated clinically and remained asymptomatic. After two months, hysteroscopy was repeated and no suspicious areas that would warrant biopsy were found. An endometrial sample was collected using Novak curettage and was subjected to histopathological analysis, from which the result was a diagnosis of hypotrophic endometrium with metaplastic areas. This patient remains under clinical follow-up evaluations and has annual ultrasound examinations. The second of these two cases consisted of endometrial adenocarcinoma in stage IaG1. This patient underwent hysterectomy, bilateral salpingoophorectomy and pelvic lymphadenectomy. No radiotherapy was administered.

## DISCUSSION

Endometrial evaluation among postmenopausal women is a topic of ongoing debate in the literature. There is a trend towards investigating intracavitary uterine lesions only in patients with postmenopausal bleeding when the endometrial thickness, as measured by ultrasound, is > 4 mm.^14-19^ Other authors have recommended systematic collection of biopsies from symptomatic patients,^20^ regardless of endometrial thickness, because of reports of cancer in patients presenting ultrasound-measured endometrial thickness ≤ 5 mm.^5,12,21^

The question is at what time, based on ultrasound measurements of endometrial thickness and the patient's history of vaginal bleeding, endometrial sample collection is indicated. In the present study, no cancer or hyperplasia was found in patients presenting endometrial thicknesses < 8 mm on ultrasound, with or without bleeding. We found that the median endometrial thickness of carcinomas and hyperplasias was considerable (17 mm) and significantly greater than the thicknesses of all other intracavitary lesions. Other authors have demonstrated similar findings, reporting mean thicknesses of carcinomas ranging from 18.2 mm to 23.05 mm.^10,17,18,22^

It is worth noting that all the benign lesions or functional endometria in our patients, including submucous myomas (which, although usually referred to as such in ultrasound reports, do not constitute real endometrial lesions), presented the same median endometrial thickness (10 mm).

With regard to which method is best for performing endometrial biopsies, Ben-Yehuda et al.^23^ defended the use of curettage as a standard diagnostic procedure. Nevertheless, based on the classic study conducted by Word et al.,^24^ it is known that this procedure fails to diagnose one in every ten lesions of the endometrial cavity. This is ascribed to the fact that, in more than half of all such cases, less than 50% of the uterine cavity is curetted, even by experienced surgeons,^25^ which can make the diagnosis difficult, especially in cases of focal uterine lesions. In addition, curettage presents higher rates of morbidity and mortality than do other methods of endometrial sampling, as well as resulting in higher hospital costs due to the need for anesthesia.^23,25^

In an attempt to improve diagnostic quality and to lower the morbidity, biopsies can be performed using less invasive procedures, such as the Vabra aspirating catheter, Pipelle or Novak curette. In comparison studies, the accuracy of traditional curettage has proven to be less than or equal to the accuracy of these other procedures,^26,27^ although these other methods produce insufficient samples in up to 28% of the cases,^28^ thus making it indispensable to also use auxiliary assessment methods.

Within this context, diagnostic hysteroscopy has been increasingly used, thereby providing evidence that allows it to be concluded that histological sample collection is not essential when the hysteroscopic appearance is normal.^15,16^ Considering the high specificity found for diagnostic hysteroscopy, our results corroborate this point.

In the present study, none of the symptomatic patients presenting normal hysteroscopy results who subsequently underwent Novak curettage biopsies were diagnosed with carcinomas or hyperplasias. This reinforces the advantage of hysteroscopy in detecting lesions in the endometrial cavity with high sensitivity (94.4%), specificity (97%) and accuracy (96.8%). As shown by Garuti et al., blind endometrial sampling is safe in excluding hyperplasias and carcinomas, thus allowing it to be assumed that no cases were actually missed.^29^

Although endometrial cancer or hyperplasia did not remain undetected in our study, this phenomenon has been described by others. Deckardt et al. found 10 cases of undetected endometrial carcinoma among 1,286 patients who underwent hysteroscopy without biopsy, and in these cases the diagnosis was made by subsequent dilatation and curettage.^21^ Perhaps if we had studied a larger sample our results would have been different. However, another possible explanation for the findings of Deckardt et al. is that they analyzed perimenopausal and postmenopausal patients together.^21^ Since functional endometrium is the pattern found around the time of the menopause, endometrial lesions possibly more often remain unrecognized than in the atrophic postmenopausal endometrium.

Another advantage found was that representative/sufficient material for analysis was acquired in 97.6% of the cases. This indicated the superiority of both hysteroscopy alone and hysteroscopy accompanied by Novak curettage biopsy over other random sampling methods used in isolation.^27,28^

The hysteroscopic impression was erroneous in one case of hyperplasia, which was taken to be a benign polyp. Nevertheless, the correct diagnosis was made when the hysteroscopy-directed biopsy was performed. This case illustrates the debate found in a recently published study of 323 hyperplasias in which the criteria for hysteroscopic diagnosis were found to be inaccurate, thereby providing justification for histopathologically assessing every irregularly lined or thickened endometrium.^30^

Among the 399 asymptomatic patients who underwent hysteroscopy, one had complex hyperplasia with atypia and another had endometrial adenocarcinoma, together accounting for only 0.5% of the examinations performed. This is consistent with the findings of Gambacciani et al.,^31^ who conducted 148 hysteroscopy procedures on asymptomatic patients with endometrial thickening and also found only one case of hyperplasia and one of adenocarcinoma. Therefore, a high number of invasive examinations were performed unnecessarily. In view of the existing evidence in the literature showing that the use of transvaginal ultrasound does not improve the diagnosis among asymptomatic patients,^8,32^ our study reinforces the opinion that performing this procedure is of questionable utility as a screening method among asymptomatic women.

## CONCLUSION

Our results suggest that hysteroscopy is valuable as a diagnostic tool for malignant/hyperplasia lesions, as well as for benign lesions, with the exception of submucous myomas, for which the sensitivity was only 52.6%.

Therefore, based upon our findings, we can conclude that the probability of identifying endometrial adenocarcinoma is minimal in asymptomatic patients presenting thin endometrium on ultrasound examination. To improve medical care among postmenopausal women, hysteroscopy should be used to obtain endometrial samples only from symptomatic patients.
